# Genome sequences of *Vibrio cholerae* strains isolated in the DRC between 2009 and 2012

**DOI:** 10.1128/mra.00827-23

**Published:** 2024-02-12

**Authors:** Alexandre Lemopoulos, Berthe Miwanda, Natália C. Drebes Dörr, Sandrine Stutzmann, Didier Bompangue, Jean-Jacques Muyembe-Tamfum, Melanie Blokesch

**Affiliations:** 1 Laboratory of Molecular Microbiology, Global Health Institute, School of Life Sciences, Ecole Polytechnique Fédérale de Lausanne (EPFL), Lausanne, Switzerland; 2 Institut National de Recherche Biomédicale (INRB), Kinshasa, Democratic Republic of Congo; 3 Département de Microbiologie, Faculté de Médecine, Université de Kinshasa, Kinshasa, Democratic Republic of Congo; Loyola University Chicago, Chicago, Illinois, USA

**Keywords:** *Vibrio cholerae*, genome sequences

## Abstract

*Vibrio cholerae* has caused seven cholera pandemics in the past two centuries. The seventh and ongoing pandemic has been particularly severe on the African continent. Here, we report long read-based genome sequences of six *V. cholerae* strains isolated in the Democratic Republic of the Congo between 2009 and 2012.

## ANNOUNCEMENT

Cholera is an acute, life-threatening diarrheal disease caused by *Vibrio cholerae* and is readily transmitted through the fecal-oral route, especially in areas with poor access to sanitation and clean drinking water (1). The ongoing seventh cholera pandemic started in 1961 in Indonesia (1) and has subsequently been introduced onto the African continent from South Asia multiple times (2, 3). Cholera is endemic in the African Great Lakes Region (4), with the Democratic Republic of the Congo (DRC) severely affected (5). Here, we announce the genome sequences of six *V. cholerae* strains originating from an unusually large epidemic that arose in the region surrounding Kisangani, later spreading westward along the Congo River during 2011–2012 (6, 7). The strains were isolated by the DRC’s Institut National de Recherche Biomédicale between 2009 and 2012 as part of their cholera surveillance program. Upon shipment to Switzerland, isolates were streaked on thiosulfate citrate bile salts agar, colony-purified, grown in lysogeny broth (LB) medium, and stored as glycerol stocks at −80°C. Following inoculation from the glycerol stock and aerobic growth in LB medium at 30°C, genomic DNA was isolated as previously outlined, employing Qiagen’s 100/G and 500/G columns in conjunction with the manufacturer’s genomic DNA buffer set (8, 9). Sequencing and assembly were performed by the Genomic Technology Facility of the University of Lausanne and followed a reported protocol (9). Briefly, high-molecular-weight DNA was sheared to obtain 10- to 15-kb fragments using a Megaruptor (Diagenode, USA). DNA (500 ng) was used to prepare a SMRTbell library using the PacBio SMRTbell Express Template Prep Kit v.2.0 (Pacific Biosciences, USA). The resulting libraries were pooled and size-selected with Ampure PacBio beads to eliminate fragments below 3 kb. The sequencing was based on v.3.0/v.3.0 chemistry and diffusion loading on a PacBio Sequel II instrument at 900-min movie length with a pre-extension time of 120 min using a SMRT cell 8M v.3. Sequence quality control, genome assembly, circularization, and rotation were performed using the protocol Microbial Assembly in SMRT Link v.10.1 with default parameters. Assembled genomes were submitted to National Center for Biotechnology Information and annotated using the Prokaryotic Genome Annotation pipeline v.6.5 (10). Information on the assembled genomes is provided in Table 1.

**TABLE 1:** Basic strain information and genome sequencing statistics

	DRC001 (#2506)	DRC052 (#2512)	DRC072 (#2516)	DRC186 (#2501)	DRC187 (#9154)	DRC193A (#1954)
Biosample	SAMN36815617	SAMN36815618	SAMN36815616	SAMN36815615	SAMN36815619	SAMN36815614
Year of isolation	2009	2012	2009	2011	2011	2011
Place of isolation (province)	Kalemie (Katanga)	Nord Kivu	Sud Kivu	Sud Kivu	Sud Kivu	Bandundu
GenBank accession numbers	JAZDAQ010000001 (chr 1) JAZDAQ010000003 (chr 2) JAZDAQ010000002 (contig 3)	CP132187 (chr 1) CP132188 (chr 2)	CP132182 (chr 1) CP132183 (chr 2)	JAVAXO010000001 (chr 1) JAVAXO010000002 (chr 2) JAVAXO010000003 (contig 3)	CP132190 (chr 1) CP132189 (chr 2)	CP132180 (chr 1) CP132181 (chr 2)
SRA^*c*^ accession numbers	SRR26435098	SRR26435097	SRR26435099	SRR26435100	SRR26435096	SRR26435101
Number of contigs	3	2	2	3	2	2
Contig sizes	2,852,572^*a*^ 1,045,468 219,361^*a*^^,*b*^	3,063,607 1,059,659	3,060,728 1,059,647	2,963,376^*a*^ 1,107,495 36,710^*a*^^,*b*^	2,959,192^*a*^ 1,107,495	3,058,770 1.045,384
Total genome size	4,117,401	4,1232,66	4,1203,75	4,107,581	4,066,687	4,104,154
% GC content	47.6% (chr 1) 47.0% (chr 2) 48.3% (contig 3)^*b*^	47.7% (chr 1) 46.9% (chr 2)	47.7% (chr 1) 46.9% (chr 2)	47.6% (chr 1) 46.3% (chr 2) 51.4% (contig 3)^*b*^	47.5% (chr 1) 46.3% (chr 2)	47.7% (chr 1) 47.0% (chr 2)
Mean coverage	140×	248×	214×	294×	277×	271×
Long read N50	13,922	16,068	15,237	16,101	16,193	18,860
Raw read count	107,377	244,481	213,984	285,783	268,327	203,915

^*a*^ Contig non-circularized.

^*b*^ Contig3 assembled separately by software but likely part of chr 1.

^*c*^ SRA, Sequence Read Archive.

Analysis of the genome sequences using Snippy v.4.6.0 (https://github.com/tseemann/snippy), Gubbins v.3.3.0 (11), RAxML v.8.12.12 (12), and Geneious v.11.0.14.1 indicated that two different strain types were isolated. The first strain type belongs to the seventh pandemic El Tor (7PET) lineage. Specifically, these strains sit within the two AFR10 sublineages recently described by Hounmanou et al. (3), with strains DRC052/DRC072/DRC193A and DRC001 belonging to clade AFR10d and AFR10e, respectively. Based on this similarity, our data support recent reports suggesting the 2011–2012 cholera epidemic in DRC was caused by a 7PET variant clonal complex (7). The second strain type (e.g., DRC186/DRC187) falls outside the 7PET clade. These non-O1 antigen strains are *ctx*- and *tcp*-negative. At this point, we cannot rule out the possibility that the sampled patients were coinfected with 7PET strains, particularly considering the limitation of strain isolation based on single colonies.

## Data Availability

The genome assemblies and raw read sequences were deposited in National Center for Biotechnology Information’s GenBank under Bioproject PRJNA1001814 and Sequence Read Archive accession numbers SRR26435096–SRR26435101.
